# Supplementary figures and images for: Vitamin A Concentration in Human Milk: A Meta-Analysis
Source: Nutrients. 2022 Nov 16;14(22):4844. doi: 10.3390/nu14224844 (PMC9699574; doi:10.3390/nu14224844)

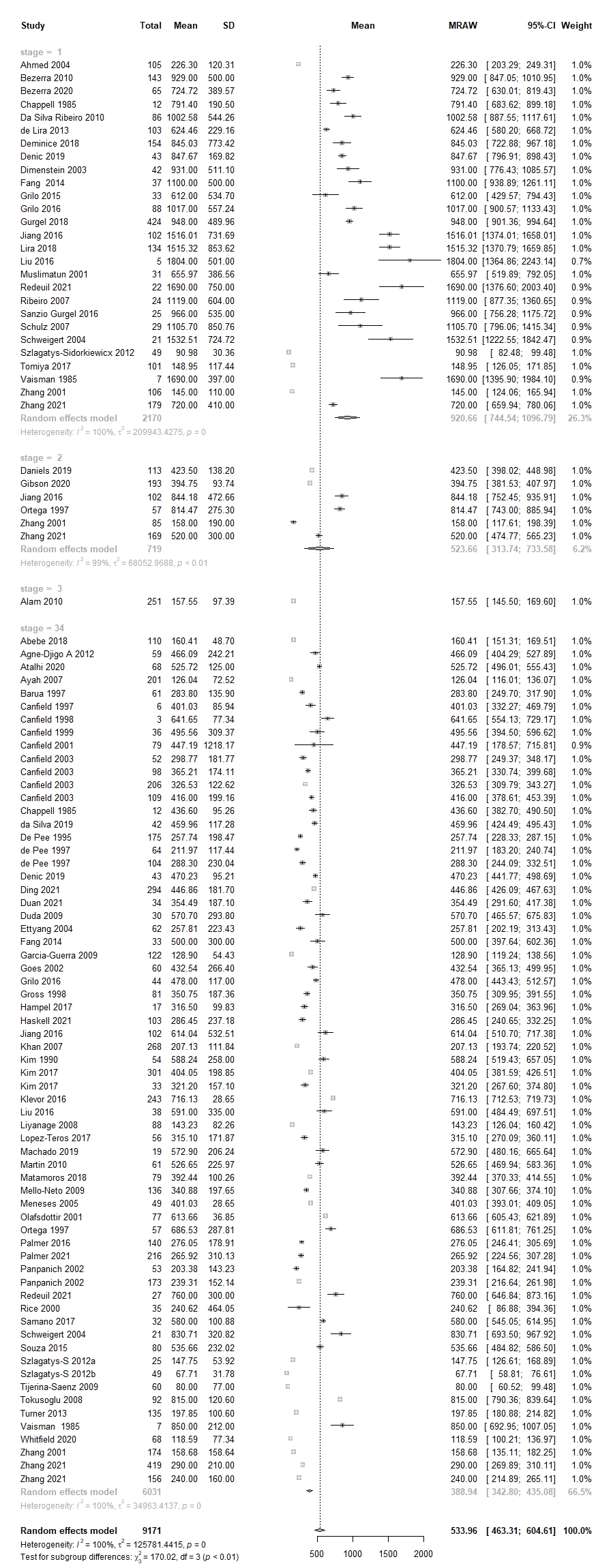

Supplement: Supplementary file 1 [file nutrients-14-04844-s001.zip › Figure S1 Forest plot of human milk VA level by 3 lactation stages.jpeg]

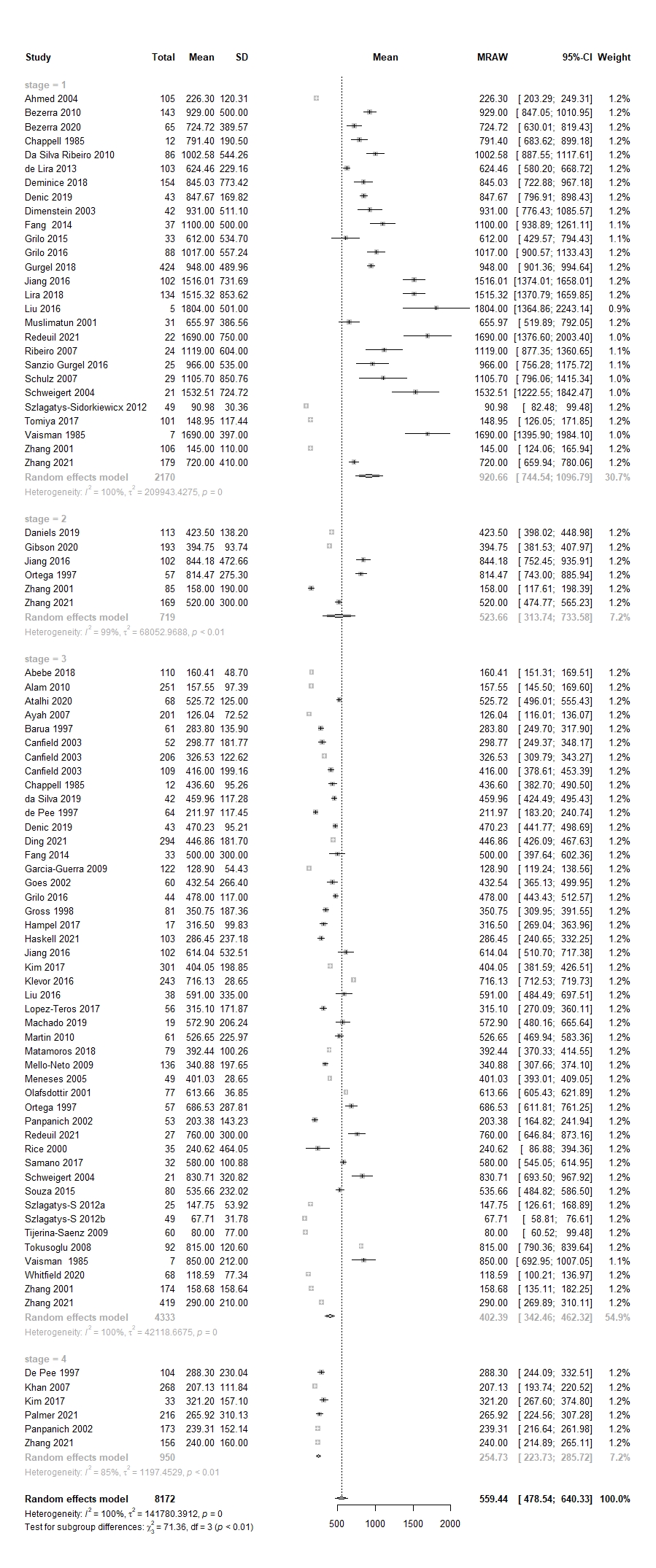

Supplement: Supplementary file 1 [file nutrients-14-04844-s001.zip › Figure S2 Forest plot of human milk VA level by 4 lacation stages.jpeg]

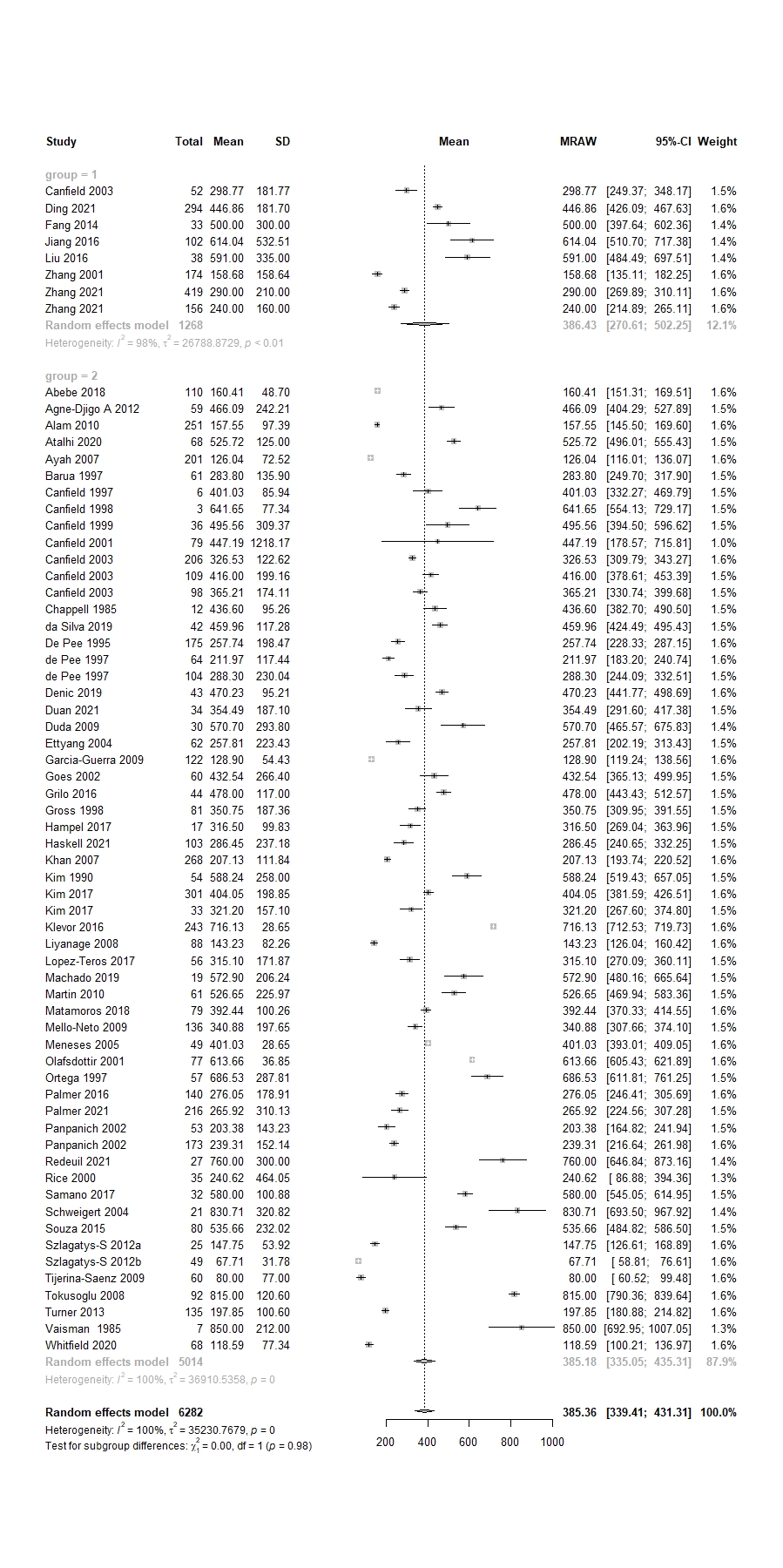

Supplement: Supplementary file 1 [file nutrients-14-04844-s001.zip › Figure S3 Forest plot of mature human milk VA level by population (Chinese vs non-Chinese).jpeg]
